# A Reflective Terahertz Point Source Meta-Sensor with Asymmetric Meta-Atoms for High-Sensitivity Bio-Sensing

**DOI:** 10.3390/bios14120568

**Published:** 2024-11-23

**Authors:** Luwei Zheng, Kazuki Hara, Hironaru Murakami, Masayoshi Tonouchi, Kazunori Serita

**Affiliations:** 1 Institute of Laser Engineering, Osaka University, Suita 565-0871, Osaka, Japank_serita@aoni.waseda.jp (K.S.); 2 Graduate School of Information, Production, and Systems, Waseda University, Kitakyusyu 808-0135, Fukuoka, Japan

**Keywords:** terahertz, terahertz biosensors, nonlinear optical crystal, point terahertz source, meta-atom, Fano resonance, reflective resonance response, DNA

## Abstract

Biosensors operating in the terahertz (THz) region are gaining substantial interest in biomedical analysis due to their significant potential for high-sensitivity trace-amount solution detection. However, progress in compact, high-sensitivity chips and methods for simple, rapid and trace-level measurements is limited by the spatial resolution of THz waves and their strong absorption in polar solvents. In this work, a compact nonlinear optical crystal (NLOC)-based reflective THz biosensor with a few arrays of asymmetrical meta-atoms was developed. A near-field point THz source was locally generated at a femtosecond-laser-irradiation spot via optical rectification, exciting only the single central meta-atom, thereby inducing Fano resonance. The reflective resonance response demonstrated dependence on several aspects, including structure asymmetricity, geometrical size, excitation point position, thickness and array-period arrangement. DNA samples were examined using 1 μL applied to an effective sensing area of 0.234 mm^2^ (484 μm × 484 μm) for performance evaluation. The developed Fano resonance sensor exhibited nearly double sensitivity compared to that of symmetrical sensors and one-gap split ring resonators. Thus, this study advances liquid-based sensing by enabling easy, rapid and trace-level measurements while also driving the development of compact and highly sensitive THz sensors for biological samples.

## 1. Introduction

Biosensing within the terahertz (THz, 0.1–10 THz) region, which lies between microwave and infrared frequencies, has captured significant attention as a highly valuable and important research topic [1,2,3,4,5]. THz waves can interact with various biological samples via their specific vibrational modes in a label-free manner, directly revealing valuable information about the composition, structure and dynamics [6,7,8,9]. Compared to X-rays and gamma rays, THz waves have the advantage of lower energy [10], which enables repeated non-destructive measurements of biological samples. Compared to microwaves, THz waves allow for high-resolution sensing because of their ultra-wide bandwidth and narrower beamwidth and save cost and power due to the efficient utility of the spectrum [11]. THz time-domain spectroscopy serves as a powerful tool with potential applications in various fields, especially in elucidating distinctive biomolecular collective motions encompassing intermolecular vibration and rotation [12,13,14,15,16,17,18]. For water-based solutions, THz spectroscopy can provide more detailed information about water molecule vibrations and the behavior of hydrogen bond networks via both waveform and spectrum, which is more useful than X-rays and microwaves, especially for studying vibrational modes and cluster structures between water molecules. Moreover, biomolecules generally exist and function in the aquatic environment. Their fingerprint features occur in the THz band, making THz spectroscopy more suitable for characterization. In addition, THz spectroscopy is well-suited for non-destructive, non-thermal and non-invasive measurement, applicable to both water-based solutions and vivo biological tissues [19,20]. Therefore, THz technology exhibits promising potential in biosensing applications. However, high-sensitivity trace-amount measurement of liquid biological samples is significantly challenging due to the limited spatial resolution restricted by the wavelength of THz waves in traditional techniques and strong absorption in polar solvents, especially in water, on which most samples are based. Therefore, it is urgent and necessary to overcome these limitations and develop more effective and compact THz sensor devices for real-world applications of THz technology.

Metamaterials, artificially designed arrays on a subwavelength scale for manipulating electromagnetic waves [21,22,23,24], have attracted increasing attention in several fields. In biosensing, metamaterials technology offers a promising approach for extracting critical biological information [25,26,27,28]. Contemporary metamaterial-based biosensors have demonstrated remarkable success in accurately detecting and measuring a wide range of biological samples, including viruses [29,30,31,32], bacteria [33,34], microorganisms [35,36], DNA [37,38,39], proteins [40,41,42] and cells [43,44,45]. However, these studies predominantly utilized dried samples. In most cases, metamaterial-based biosensors operate in the transmission mode [31,41,43,44,46,47,48,49,50]. Biological tissues and specimens generally survive and function normally in a liquid environment, in which transmission-mode biosensors are not effective due to the strong absorption, complicated optical refraction and scattering arising from the irregular shape of liquid samples. Using microfluidic technology is one of the solutions to this problem, where liquid samples are confined in a well-developed microfluidic channel for effective interaction with THz waves [51,52,53,54,55]. A liquid film with a suitable thickness is required for reliable measurement in the transmission mode. In comparison, biosensors operating in the reflection mode exhibit superior behavior with easy and rapid measurement of liquid-type samples [56,57,58]. No special requirements for the shape of liquid samples are imposed, and sample addition procedures are simplified.

The spatial resolution in conventional THz systems is restricted by the diffraction limit determined by the wavelength of THz waves, which makes subwavelength-scale measurement significantly challenging. However, near-field technology [52,53,54] serves as a remarkable approach to overcome this problem. Compared to far-field technology [41,51,59,60], the divergence of THz beams is significantly reduced in the near-field region, leading to a significantly smaller effective sensing area. Therefore, small quantities of samples at the level of a few microliters are required, which is highly favorable for achieving trace-amount measurements and compact biosensor design. Moreover, the sensitivity is also expected to be higher with improved sensing performance. It is well known that metamaterials have different resonance modes, such as LC, dipole, quadruple and Fano resonance [23,61,62,63]. Among these, Fano resonance, generally obtained by symmetry-breaking of the metamaterial structure [63], exhibits relatively superior performance and has been applied to real applications for achieving higher sensitivity [64,65,66,67,68,69,70]. Considering characteristic investigation of resonance response and sensing applications, Fano resonance is generally studied from the far-field perspective in the transmission mode. Few reports of Fano resonance response in the near-field region and in reflection mode are available, but which is expected to provide us with meaningful and necessary information for further exploration.

In this study, highly sensitive near-field THz meta-sensors equipped with a few arrays of asymmetrical meta-atoms operating in the reflection mode were developed for trace-amount measurements of liquid biological samples easily and rapidly. A near-field point THz source with a diameter of ~*ϕ* 20 μm, which significantly reduces the effective sensing area and required sample quantities for achieving trace-amount sensing, was locally generated via optical rectification with laser pulses focused on a nonlinear optical crystal (NLOC), GaAs. Only the single central meta-atom is excited by the local point THz source. Initially, resonance responses of three distinct symmetric/asymmetric meta-structures were compared to identify the optimal sensing mode. By shifting one gap from the center to the side, the symmetry of the meta-atom structure was intentionally disrupted to achieve a potentially improved resonance mode with higher sensitivity. Both the calculated and measured reflectance spectra of the double-gap asymmetrical meta-sensor were analyzed to investigate its sensing characteristics, including the Q-factor and sensitivity to changes in the refractive index of the surrounding dielectric environment. The resonance response with superior sensing performance was identified for further exploration and application. Subsequently, a series of meta-structures were developed to study the dependence of the resonance response dependence on different parameters, including structural parameters, excitation point, thickness, structural asymmetricity and array-period arrangement to explore the tunability of the reflective resonance response. In addition, the developed meta-sensor operates in the reflection mode, aiming for sensing liquid biological samples. Trace-amount double-strand DNA (dsDNA) and single-strand DNA (ssDNA) were used as test samples, which could be directly dropped onto the meta-sensor for easy and rapid operation. This work is of significance for developing trace-amount sensing methods easily and rapidly and compact, highly sensitive meta-sensor chips working in the reflection mode for real-life applications.

## 2. Materials and Methods

### 2.1. Meta-Atom Structure Fabrication and Simulation Settings

**Figure 1:**
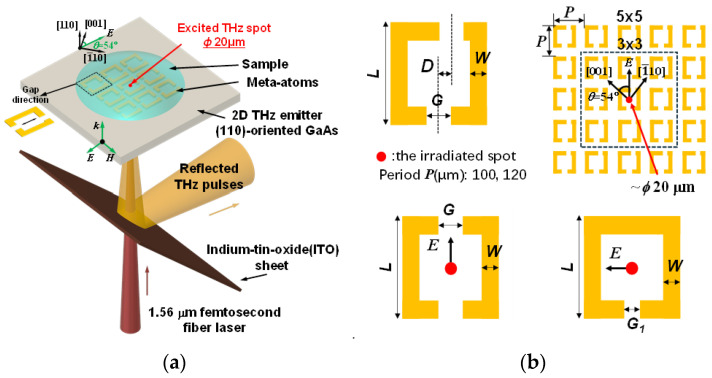
(**a**) Schematic of the experimental setup around the sample. (**b**) The geometric parameters and array-period arrangement of the three types of meta-atom structures developed. For the double-gap asymmetrical structure, the overall size (***L***) of one single meta-atom is 84 µm × 84 µm with a linewidth (***W***) of 10 µm and gaps (***G***) of 20 µm. One gap is shifted to one side with offset distance (***D***), which determines the asymmetry of the meta-structures. The meta-atom arrays are arranged in 3 × 3 and 5 × 5 patterns with periods of 100 µm and 120 µm, respectively. The gap direction is at an angle of 54 degrees to the [001] crystal orientation of the GaAs substrate. The laser irradiation spot of ~*ϕ* 20 µm is at the center of the central meta-atom. For comparison, two additional meta-atom structures were developed, as shown at the bottom of the diagram: double-gap and single-gap symmetrical structures. In the double-gap structures, the electric field aligns parallel to the gap direction, while in the single-gap structure, the electric field is oriented perpendicular to the gap direction, as indicated by the black arrows. The gap size (***G*_1_**) of the single-gap structure is 5 µm.

A series of meta-atom structures with varying array numbers, periods and offset distances, as shown in Figure 1b, were prepared to investigate their reflective properties. These structures were fabricated on a 500 µm thick, (110)-oriented GaAs substrate, known for its nonlinear optical properties conducive to THz generation via optical rectification [71], which depends on the polarization of the incident laser [52,72]. The fabrication process started with the deposition of a 1 nm thick titanium adhesion layer onto the GaAs substrate, followed by sputtering of a 200 nm thick gold layer using conventional photolithography and radio-frequency sputtering techniques. Appropriate structural parameters were carefully selected to achieve the resonance response in the studied THz region. After fabrication, the meta-atom structures were inspected under an optical microscope to check for any fabrication defects, ensuring that only defect-free structures were used for actual measurements.

The designed geometrical parameters and arrangements of the meta-atoms were employed to calculate their resonance responses using the finite difference time domain (FDTD) method with commercial software (ANSYS Lumerical FDTD 2024 R1.2). Typical calculated reflectance spectra for three types of meta-structures can be found in the Supplementary Materials (Figure S2 in the Supplementary Materials). For basic settings, the material of the meta-atoms was modeled as a perfect electrical conductor. GaAs was assigned a thickness of 500 μm and a refractive index of 3.59. A point THz source was placed at the center of the central meta-atom, positioned 50 μm below the upper surface of the substrate. This THz source was configured as a Gaussian type with an amplitude of 100, covering a wavelength range from 333 μm to 2998 μm. The simulation was conducted at a temperature of 300 K, and perfectly matched layer boundary conditions were used. The relevant parameters in the simulation were adjusted accordingly when studying the resonance response dependencies.

### 2.2. Experimental Setup

A near-field point THz source reflection system [58,73] was employed, which uses a femtosecond fiber laser (FemtoFiber pro IR, TOPTICA Photonics AG, (Munich, Germany): maximum power of 400 mW, wavelength of 1.56 μm, pulse width of 100 fs, and repetition rate of 80 MHz) as the optical source. The emitted laser beam was split into pump and probe beams by a beam splitter. The pump beam was modulated by an optical chopper, providing a reference frequency for the lock-in amplifier and eventually focused on the GaAs substrate with the meta-atoms as the THz emitter. A point THz source with a size of 20 μm was locally generated at the laser irradiation spot via optical rectification. The generated THz pulses interacted with one of the meta-atoms, were then reflected at the upper surface of the GaAs and a 175 μm thick indium tin oxide-coated PET sheet, and finally detected by a spiral-shaped low-temperature-grown GaAs photoconductive antenna. Moreover, the detector was excited by the probe beam, with the wavelength converted from 1560 nm to 780 nm by a periodically poled LiNbO_3_ nonlinear crystal via second-harmonic generation. The amplitudes and phases of the THz pulses were monitored by changing the position of the time-delay stage, and time-domain waveforms were obtained. Subsequently, the frequency spectrum was calculated by Fourier transform. The reflectance *R* of the meta-atoms was calculated by $R\left(\omega \right)=R_{R,MM}\left(\omega \right)/R_{R,ref}\left(\omega \right)$, where $R_{R,MM}\left(\omega \right)$ and $R_{R,ref}\left(\omega \right)$ are the frequency-dependent reflected THz amplitudes with and without meta-atom structures, respectively. Further, *ω* represents the frequency of the generated THz wave. When the sample is dropped on the meta-sensor, $R_{R,MM}\left(\omega \right)$ is replaced by $R_{R,S}\left(\omega \right)$, which is the frequency-dependent THz amplitude reflected by the sample. To excite the center of the central meta-atom with the point THz source, the reflected laser image was visualized using a photodiode detector (Figure S1 in the Supplementary Materials).

## 3. Results and Discussion

### 3.1. Terahertz Reflective Meta-Sensors

Our developed meta-sensors operated in the reflection mode and interacted with samples in a near-field manner. Figure 1a shows a schematic of the experimental setup around the sample. A point THz source with a diameter of ~*ϕ* 20 μm was locally generated to excite the central meta-atom. The excitation occurred under the condition that the electric field was aligned with the gap direction, allowing neighboring meta-atoms to be electrically coupled via the coupling effect [52]. The liquid biological sample was directly applied onto the meta-structure surface, interacting with the meta-sensor and influencing its resonance response due to changes in the surrounding dielectric environment. The THz signal containing the sample information was obtained as described above in Section 2.2. Figure 1b displays the geometric parameters and array-period arrangement of the developed meta-structures. The asymmetrical elementary unit is a double-gap split ring resonator with a size of 84 μm × 84 μm with a linewidth of 10 μm and gaps of 20 μm. One of the gaps is shifted parallel to the gap direction by an offset distance ***D*** relative to the other gap, creating the asymmetry. The meta-structures were arranged in different arrays of 3 × 3 and 5 × 5 and different periods of 100 μm and 120 μm, respectively. Additionally, two symmetrical structures, one with two gaps (***D*** = 0) and the other with a single gap, which are known as typical LC resonance-type sprit ring resonators, were also studied for comparison. By breaking the symmetry in the double-gap structure, enhanced resonance performance was expected. To obtain the maximum THz generation efficiency to excite the meta-atoms, the direction of the excitation electric field was oriented at an angle of 54 degrees to the [001] crystal orientation of the GaAs substrate [52]. The meta-structures were fabricated on the GaAs substrate in a specific orientation, with the electric field parallel and perpendicular to the gap direction in the double-gap and the single-gap structures, respectively.

### 3.2. Resonance Modes in the Reflection Mode

**Figure 2:**
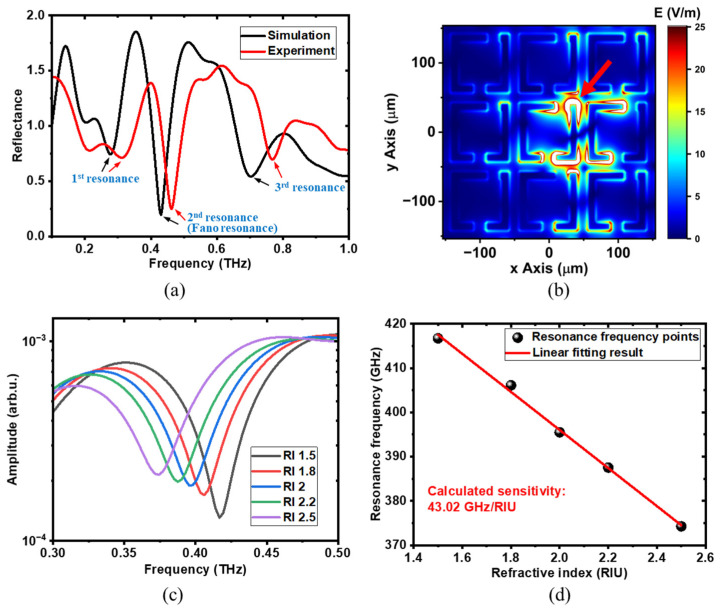
Reflective resonance analysis with the meta-structure arrayed in a 5 × 5 pattern with a period of 100 µm and gap offset distance of 20 µm. (**a**) The calculated and measured reflectance spectra. There main resonances are observed: 1st resonance, 2nd resonance (Fano resonance), and 3rd resonance, which are indicated by black and red arrows for simulation and experiment, respectively. (**b**) The corresponding electric field distribution of the 2nd resonance (Fano resonance) at 0.425THz. A strong electric filed coupling region was observed around the shorter rod, as the red arrow indicates. (**c**) The calculated 2nd resonance frequency spectra of the meta-structure immersed in materials with different refractive indices. (**d**) The calculated resonance frequency versus refractive index with a linear fitting result. The evaluated sensitivity is 43.02 GHz/RIU.

To obtain the highest resonance response, comparative studies were conducted. Figure 2 shows the reflective resonance characteristics of the 5 × 5 arrays of the meta-structure with a meta-atom period of 100 μm and gap offset distance of 20 μm. Initially, the calculated and measured reflectance spectra were compared, as shown in Figure 2a. Notably, in some frequency regions, the reflectance values exceed 1 due to the effect of resonance enhancement [49]. As the definition of reflectance mentioned above demonstrates, the reference signal is the reflected THz signal without meta-atom structures. When meta-atom structures are involved, their resonance response modulates the spectrum, resulting in response enhancement and attenuation effects at different frequency components. In the case of response enhancement, the calculated reflectance values are greater than 1 because these frequency components have become stronger compared to the corresponding reference frequency components. From the spectra, three distinct resonance peaks, labeled as the 1st, 2nd and 3rd resonances, were observed. In the experimental results, the resonance frequencies of all three resonances shifted to higher values compared to the simulated results. The reason behind this frequency shift will be discussed in detail later. In the calculated reflectance spectrum, each resonance appears at approximately 0.275 THz, 0.425 THz and 0.7 THz, with the Q-factor calculated to be 6.13, 8.9 and 8.58, respectively. The Q-factor can be calculated by the formula $Q=f_{r}/∆f$, where *f_r_* is the resonant frequency and ∆*f* is the full width at half maximum. In the measured reflectance spectrum, resonances were observed at approximately 0.31 THz, 0.46 THz and 0.77 THz, with Q-factors calculated to be 6.25, 10.48 and 13.25, respectively. Among these, the 2nd and 3rd resonances exhibited large Q-factors, surpassing the value of 6 in the transmission mode [53] and indicating superior sensing capabilities. Although the 3rd resonance also showed a relatively large Q-factor, it demonstrated poor sensitivity to changes in the external dielectric environment, making it less suitable for sensing applications (Figure S4 in the Supplementary Materials). Figure 2b displays the electric field distribution at 0.425 THz during the 2nd resonance, which is the Fano resonance obtained by using the asymmetrical meta-structure (Figure S2 in the Supplementary Materials). Compared to the electric field distribution of the double-gap symmetrical meta-structure described in our previous work [58], a distinct electric field distribution and coupling associated with Fano resonance were observed. In this scenario, only the central single meta-atom was directly excited by the electric field component of the THz waves, while its neighboring meta-atoms were subsequently excited through a coupling effect. As indicated by the red arrow in Figure 2b, a significantly stronger electric field coupling region was observed around the shorter rod of the meta-atom. This suggests strong interactions between liquid samples and the THz electric field, highlighting the excellent sensing capability at this resonance. A similar electric field distribution has been noted in the transmission mode [53]. In contrast, the electric field distributions in the coupling region for the 1st and 3rd resonances are significantly weaker compared to that in the 2nd resonance (Figure S4 in the Supplementary Materials), resulting in reduced sensitivity at these two resonances. The floating capacitance in this region plays a major role in the coupling effect and is primarily responsible for sensing performance. As the liquid samples modify the floating capacitance around the shorter rod, the resonance response of the meta-atoms changes, thereby enabling the detection of liquid biological samples. To assess its sensing performance, further simulations were conducted by varying the refractive index of the material in which the meta-structure is immersed, as shown in Figure 2c. The simulation geometry configuration can be found in the Supplementary Materials (Figure S3 in the Supplementary Materials). As the refractive index increases, the resonance frequency gradually shifts to lower values. This is because materials with higher refractive indices increase the floating capacitance between the meta-atoms, leading to changes in the coupling intensity. The refractive index dependence of the 2nd resonance is illustrated in Figure 2d, where the black dots represent the extracted resonance frequencies from Figure 2c, and the red line is the linear fit, yielding a calculated sensitivity of 43.02 GHz/RIU. The linear fitting equation used is RF=s·RI+a, where *RF* and *RI* are the datasets of the resonance frequency and refractive index, respectively; *a* is the fitted intercept. In comparison, the sensitivities of the 1st and 3rd resonances were evaluated at 20.14 GHz/RIU and 11 GHz/RIU, respectively (Figure S4 in the Supplementary Materials). The 2nd resonance exhibited the highest sensitivity and superior sensing performance, making it more favorable for sensing liquid biological samples. In addition, the refractive index dependence of two other symmetrical structures—one with two gaps and the other with a single gap—were also evaluated, which were 27.77 GHz/RIU [58] and 20.84 GHz/RIU (Figure S5 in the Supplementary Materials), respectively. Overall, the 2nd resonance (Fano resonance) peak of the double-gap asymmetric meta-atom structure demonstrates the highest sensitivity to refractive index changes, making it the most promising for sensing applications.

### 3.3. Resonance Response Dependence Analysis

#### 3.3.1. Geometric Size Dependences

**Figure 3:**
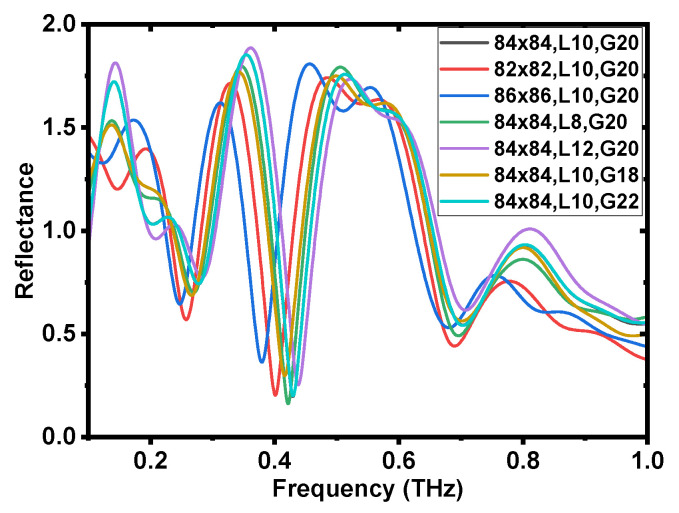
Geometric size dependence. The simulated reflectance spectra of the meta-atom structures with different geometric parameters. The overall dimensions (***L***) of each meta-atom were set as 82 μm × 82 μm, 84 μm × 84 μm, and 86 μm × 86 μm, respectively. The linewidth (***W***) was defined as 8 μm, 10 μm, and 12 μm, and the gap sizes (***G***) were set at 18 μm, 20 μm, and 22 μm. These three parameters were varied in combination, resulting in a total of seven different cases.

For metamaterial structures, the resonance response is influenced by several factors, demonstrating its tunability and accounting for the discrepancies between the simulated and measured results shown in Figure 2a. It is well known that resonance response characteristics are highly sensitive to geometric sizes. Figure 3 presents the calculated reflectance spectra of the geometric size dependence. The overall size (***L***) of a single meta-atom was set to three configurations: 82 μm × 82 μm, 84 μm × 84 μm and 86 μm × 86 μm. Similarly, three different line widths (***W***) were considered, 8 μm, 10 μm and 12 μm, along with three gap sizes (***G***): 18 μm, 20 μm and 22 μm. Each case involved altering only one parameter relative to the original design, forming a total of seven combinations. From the calculated spectra, noticeable shifts in the resonance frequency were observed regardless of which parameter was altered. These three parameters can sensitively change the electric field distribution and, thus, the coupling state, resulting in shifts in the resonance frequency. In actual measurements, the geometry of the fabricated meta-atom structure generally deviates from the designed parameters used in the simulation, which is a key factor contributing to the discrepancy between the simulation and experimental spectrum shown in Figure 2a (Figure S6 in the Supplementary Materials).

#### 3.3.2. Position Dependence of the Excitation of Point THz Sources

**Figure 4:**
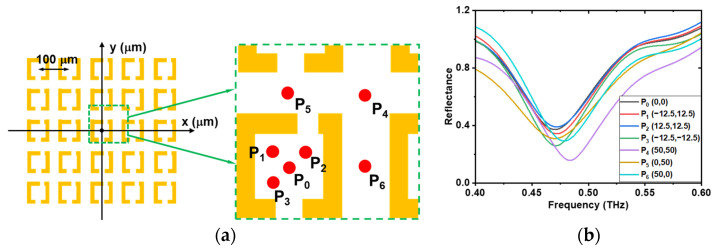
Position dependence of the excitation of point THz sources. (**a**) Schematic of laser excitation point positions: P_0_(0, 0), P_1_(−12.5, 12.5), P_2_(12.5, 12.5), P_3_(−12.5, −12.5), P_4_(50, 50), P_5_(0, 50), and P_6_(50, 0). (**b**) The corresponding reflectance spectra at each laser excitation point position.

In our case, the meta-structure was excited in the near field using a point THz source. The position of the point THz source directly affects the excitation state of the meta-atoms, thereby affecting the resonance response. Figure 4 displays the reflectance response depending on the excitation positions of the THz sources. The same meta-atom structure as that shown in Figure 2, arranged in the 5 × 5 arrays of meta-atoms with a meta-atom period of 100 μm and a gap offset distance of 20 μm, was selected as the example structure. Seven different excitation positions of the THz point sources were examined, as shown in Figure 4a. The origin P_0_(0, 0) is set at the center of the central meta-atom, and the XY coordinates are adjusted to match the scale of the meta-atom array. Four points—P_0_(0,0), P_1_(−12.5, 12.5), P_2_(12.5, 12.5) and P_3_(−12.5, −12.5)—were located within the central meta-atom, while three points—P_4_(50, 50), P_5_(0, 50) and P_6_(50, 0)—were positioned between meta-atoms. The size of the red dot was the same as that of the THz point source. The corresponding reflectance spectra for each excitation point are displayed in Figure 4b, where clear shifts in the resonance frequency and changes in amplitude were observed. From the experimental results, the position of the resonance response remained largely unaffected by changes in the excitation point within the meta-atom unit, indicating minimal dependence on its precise location. However, when the laser was focused outside the meta-atom unit, slight reductions in the Q-factor were observed. For optimal resonance and sensitivity, it is preferable to position the excitation point within the meta-atom unit. Since the excitation occurs in the near-field, the resonance response is more sensitive to structural and excitation variations compared to that in the far-field technique. The resonance response is determined by the coupling between meta-atoms. Similar results were found in the simulation (Figure S7 in the Supplementary Materials). In the experiments, positioning of the focused laser beam spot was achieved by laser reflection imaging, which introduced positioning errors and also contributed to the above-mentioned discrepancy between the calculated and measured results.

#### 3.3.3. Thickness Dependence

**Figure 5:**
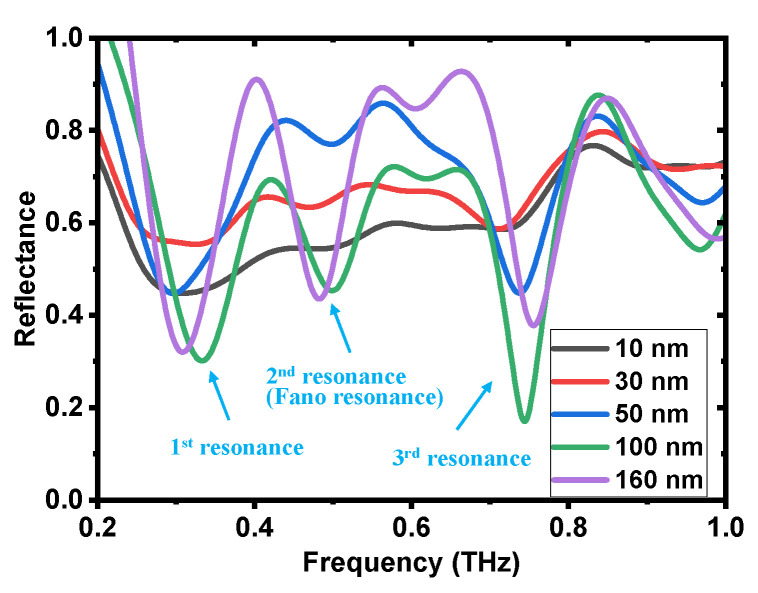
Thickness dependence. The reflectance spectra of the meta-atom structures with different thickness from 10 nm to 160 nm.

The thickness of the meta-atoms is another important parameter affecting the resonance response. Figure 5 demonstrates the measured thickness dependence, including the reflectance and transmittance spectra (Figure S8 in the Supplementary Materials). The thickness of the meta-structure ranged from 10 nm to 160 nm, which was controlled by varying the sputtering time. As the thickness decreases, the intensity of the resonance response becomes progressively weaker in both the reflection and transmission modes. This is because as the structure’s thickness decreases, the equivalent resistance of the structure is enhanced, resulting in weaker currents generated. This reduction in current lowers the strength of the electric field in the coupling region, diminishing the coupling effect and ultimately weakening the resonance response [74]. With a thickness of 160 nm, a strong resonance peak can be observed. As the thickness decreases to 10 nm, the Fano resonance nearly vanishes. For sensing applications, adequate resonance strength is essential. It is evident that a thickness over 50 nm yields a significant resonance response, implying that there is a thickness limitation for strong resonance. In addition, as the thickness changes, a slight resonance frequency shift can also be observed, which is also one of the reasons for the discrepancies between the simulated and experimental results.

#### 3.3.4. Distance, Number and Period Dependences

**Figure 6:**
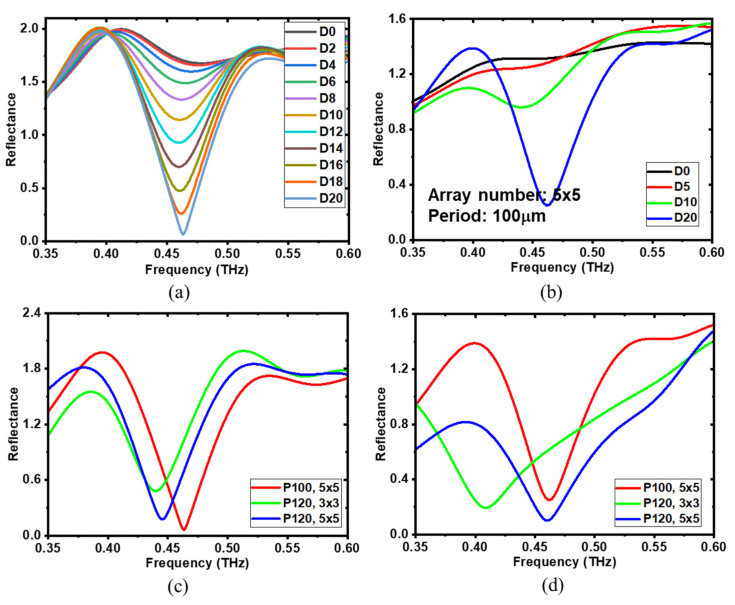
Gap offset distance, array number and period dependences of the developed meta structures. The calculated (**a**,**b**) measured reflectance spectra of meta-structures with changing offset distance from 0 µm to 20 µm with a step of 2 µm and 5 µm, respectively. The array number and period are fixed at 5 × 5 and 100 µm, respectively. The calculated (**c**) and measured (**d**) reflectance spectra of meta-structures with different arrays and periods.

To further explore the reflective characteristics and tunability of the resonance response, several design parameters were investigated, including the gap offset distance, array number and period. Figure 6a demonstrates the calculated reflectance spectra for the gap offset distance ranging from 0 μm to 20 μm in 2 μm increments. The array number and period are fixed at 5 × 5 and 100 μm, respectively. Notably, as the gap offset distance increases, the 2nd resonance grows progressively stronger. Structural symmetry breaking leads to a more concentrated electric field distribution, as indicated in the coupling region around the shorter rod of the meta-structure in Figure 2b. As the asymmetry increases and the shorter rod shortens, the electric field in that region intensifies, ultimately enhancing the resonance response. Figure 6b displays the corresponding measured results with changing gap offset distance from 0 μm to 20 μm with a step of 5 μm. From the experimental results, resonance peaks were only observed at gap offset distances of 10 μm and 20 μm, with a significantly stronger resonance response at 20 μm, which agreed well with the simulation results. The absence of resonance peaks at 0 μm and 5 μm is attributed to the weakness of the resonance response under these conditions. Consistent with the simulation, the resonance response was weak when the gap offset distance was less than 6 μm.

The array number and period are the two most significant parameters affecting the coupling effect between meta-atoms. To investigate their impact, array numbers 3 × 3 and 5 × 5 and periods of 100 μm and 120 μm were selected to examine the array number and period dependences. The calculated and measured results are shown in Figure 6c and Figure 6d, respectively. The same tendencies were observed in both the simulation and experimental results. When the period was fixed at 120 μm, increasing the array number from 3 × 3 to 5 × 5 shifted the resonant frequency to higher values. Additionally, the resonance peak deepened, indicating a stronger resonance response. This is because a larger array number results in more coupling interactions between the meta-atoms, which enhances the resonance effect [53]. Note that this enhancement is limited by the local excitation of the THz point source. As the distance from the central excited meta-atom increases, the outer meta-atoms are less excited by the coupling effect until the excitation fades. As shown in the electric field distribution in Figure 2b, the outer meta-atoms are significantly less affected compared to the inner ones, demonstrating the excitation limitation. For the meta-structure arranged in a 3 × 3 array with a period of 120 μm, a larger discrepancy between the simulation and experimental results was observed compared to that in the 5 × 5 patterned structures. This is because the 3 × 3 array has fewer meta-atoms, which makes it more sensitive to the parameters discussed above. Additionally, our measurements were conducted in a near-field approximation, making it more sensitive compared to the far-field case, which shows almost no dependence on the array number due to the presence of numerous meta-atoms. When the array number was fixed at 5 × 5, reducing the period from 120 μm to 100 μm shifted the resonant frequency to higher values, and the resonance response was enhanced. A smaller period results in a shorter distance between meta-atoms, facilitating stronger coupling interactions and a more pronounced resonance response. The simulation and experiment yielded similar results, with only slight differences caused by the differences between the simulation and experimental conditions, as analyzed above. Overall, both the calculated and experimental findings confirm that the reflective resonance response is highly dependent on the examined parameters, gap offset distance ***D***, array number and period, exhibiting good tunability in designing suitable meta-atom-based THz sensor chips for real applications, in which meta-atom structures and biological samples have the same eigen-resonant frequency.

### 3.4. dsDNA and ssDNA Measurement

**Figure 7:**
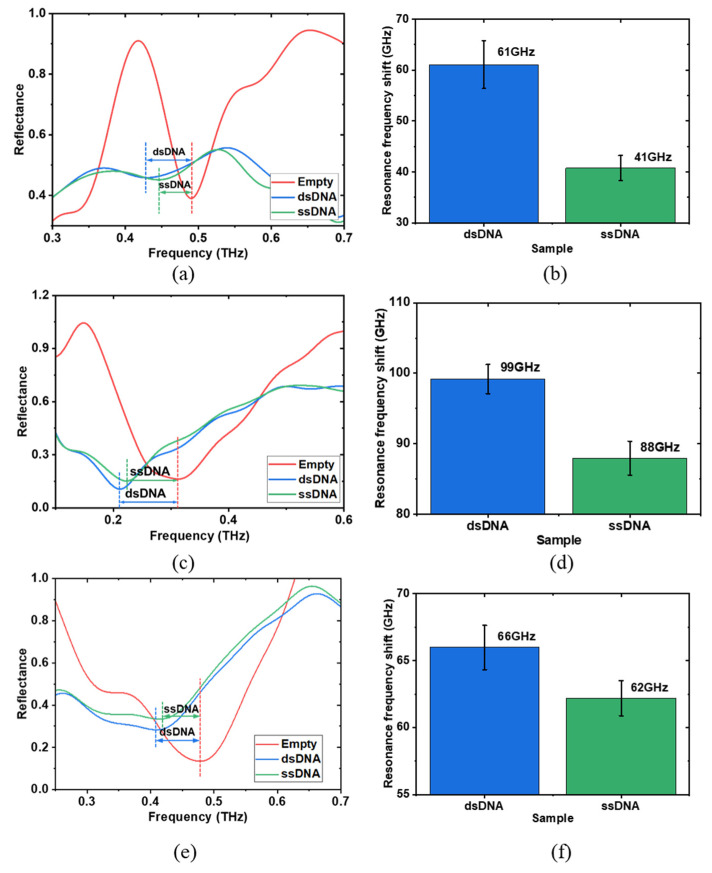
dsDNA and ssDNA measurements. Reflectance spectra (**a**,**c**,**e**) of the meta-sensor with and without dsDNA and ssDNA in a single measurement. Average resonance frequency shifts (**b**,**d**,**f**) of dsDNA and ssDNA of ten measurements. (**a**,**b**) are the results of double-gap asymmetrical meta structure. (**c**,**d**) are the results of double-gap symmetrical meta structure. (**e**,**f**) are the results of single-gap symmetrical meta structure. The concentration of the measured dsDNA and ssDNA are 100 µg/mL. The array number and period of the three meta-structures are set as 5 × 5 and 100 µm, respectively.

To evaluate the sensing performance for real biological applications, dsDNA and ssDNA were examined. DNA samples were procured from Promega Corporation, with initial concentrations of 584 μg/mL for dsDNA and 100 μg/mL for ssDNA. To ensure a precise comparison, the dsDNA solution was diluted to match the concentration of the ssDNA. The sensing performances of the double-gap asymmetrical meta-structure were estimated alongside two other meta-structures: double-gap symmetrical and single-gap symmetrical meta-structures, as shown in Figure 1b and Figure 7. For all three meta structures, the array number and period were set to 5 × 5 and 100 μm, providing a sensing area of 0.234 mm^2^ (484 μm × 484 μm), which requires only a small volume of sample solutions. Before the DNA measurement, resonance frequency dependence on ultra-pure water drying process was examined to investigate the effect of water evaporation using the double-gap asymmetrical meta-structure (Figure S9 in the Supplementary Materials). During the experiments, 1 μL of DNA solution, sufficient to fully cover the sensing area, was carefully applied using a micro-syringe (Sartorius Biohit, 0.1-3 μL (Transcat, Rochester, NY, USA)). The DNA solution altered the dielectric environment around the meta-atoms, affecting their resonance response, with biological information encoded in the reflectance spectrum. By analyzing the resonance frequency shift, the dsDNA and ssDNA samples, which have different refractive indices, could be distinguished. After each deposition of the DNA solution, the reflected THz signal was immediately measured once, and the DNA solution was subsequently cleaned with ultra-pure water, making the meta structures reusable for further liquid sample testing. Each measurement was repeated ten times, and the average reflectance was calculated. Figure 7a shows the reflectance spectra for the double-gap asymmetrical meta-structure during a single measurement. The resonance frequency was observed at approximately 0.49 THz when no DNA samples were present on the meta structure and shifted to lower values after dropping the DNA samples. The resonance peaks appeared at approximately 0.425 THz and 0.445 THz for dsDNA and ssDNA, respectively. Notably, the resonance frequency shift of dsDNA was higher than that of ssDNA due to its larger refractive index [75], which is consistent with the calculation results of the refractive index dependence shown in Figure 2c. In our measurement, the DNA samples were directly dropped, which enables easier and more rapid detection of liquid biological samples in the THz region. To increase the reliability of the obtained data, each sample was measured ten times, and the results were averaged. Figure 7b shows the average resonance frequency shift for dsDNA and ssDNA, which were 61 GHz and 41 GHz, respectively, with standard deviations of 4.6 GHz and 2.4 GHz, respectively. The disparity of 20 GHz in resonance frequency shift enables differentiating dsDNA and ssDNA based on their distinct refractive indices. Actually, metamaterial-based biosensors can also demonstrate sensitivity to changes in DNA concentration through shifts in resonance frequency [76].

Figure 7c and Figure 7d display the corresponding results for the double-gap symmetrical meta-structure, where the resonance frequency shifts were 99 GHz for dsDNA and 88 GHz for ssDNA, with standard deviations of 2.1 GHz and 2.4 GHz, respectively. Figure 7e and Figure 7f show the corresponding results of the single-gap symmetrical meta-structure, with resonance frequency shifts of 66 GHz for dsDNA and 62 GHz for ssDNA and standard deviations of 1.6 GHz and 1.3 GHz, respectively. The resonance frequency shift differences of dsDNA and ssDNA for these two meta-structures were 11 GHz and 4 GHz, respectively. The 2nd resonance of the double-gap asymmetrical meta structure exhibited the largest distinction between dsDNA and ssDNA, offering a sensitivity twice that of the double-gap symmetrical structure and five times that of the single-gap asymmetrical structure, demonstrating its superior sensing performance with the highest sensitivity. The higher sensitivity of the asymmetrical double-gap meta-structure compared to the symmetrical double-gap structure is attributed to the asymmetry, which results in a more concentrated electric field distribution. In comparison to the symmetrical single-gap structure, the enhanced sensitivity is due to the presence of two gap regions in the double-gap structures. This increases the area where the electric field is distributed and enhances the coupling effect between the meta-atoms, ultimately leading to higher sensitivity.

## 4. Conclusions

We developed a reflective THz meta-sensor with a few arrays of asymmetric meta-atoms for highly sensitive, trace-amount sensing using the Fano resonance mode. A series of double-gap asymmetrical meta-atom structures were developed and fabricated, where only the single central meta-atom was excited by a locally generated point THz source with a diameter of ~*ϕ* 20 μm in the near-field region. Neighboring meta-atoms were then stimulated through the coupling effect. In the structure featuring a 5 × 5 array with a period of 100 μm, three distinct reflective resonance peaks were observed. Their sensing performance, in terms of the Q-factor and sensitivity to changes in the surrounding refractive index, was evaluated. Compared to the symmetrical double-gap and single-gap meta-atoms, which induced typical LC resonance modes, the Fano resonance exhibited superior performance. This resonance was affected by several factors, including geometric size, excitation point position, thickness, structural asymmetricity and array-period arrangement, indicating its good tunability. Apparent Fano resonance occurs only when the structure is sufficiently thick, and the asymmetricity is sufficiently strong. To estimate the sensing performance of the meta-sensor with an effective sensing area of 0.234 mm^2^, dsDNA and ssDNA were examined. Notable resonance frequency shifts of 61 GHz and 41 GHz were observed for both dsDNA and ssDNA, respectively. For comparison, the sensing performance of two additional structures, double-gap and single-gap symmetrical meta-atoms, were also evaluated. However, they showed lower sensitivity and less pronounced frequency shifts, making them less effective for detecting DNA samples. The Fano resonance resulting from structural symmetry breaking exhibited enhanced sensitivity, making it a promising approach for sensing. This work should aid in developing trace-amount sensing methodologies in the reflection mode with the advantages of easy and rapid operation for liquid biological samples and accelerating the design of compact, highly sensitive THz biosensors.

## Data Availability

The raw data supporting the conclusions of this article will be made available by the authors upon request.

## Supplementary Materials

The following supporting information can be downloaded at: https://www.mdpi.com/article/10.3390/bios14120568/s1, Figure S1: Laser reflection imaging results of the double-gap asymmetrical meta-structure arranged in a 5 × 5 array with a period of 100 μm. Figure S2: Reflectance spectra of double-gap symmetrical (***D*** = 0) and asymmetrical (***D*** = 20 μm) meta-structures. Figure S3: Simulation geometry configuration. Figure S4: The resonance mode analysis of the 1st and 3rd resonances.; Figure S5: Refractive index dependence of the single-gap symmetrical meta structure; Figure S6: Optical image of the fabricated asymmetrical meta-atoms; Figure S7: The calculated reflectance spectra at each laser excitation point position; Figure S8: Thickness dependence in transmission mode. Figure S9: Resonance frequency dependence on ultra-pure water drying process. References [53,68] are cited in Supplementary Materials.
