# Supplementary material for: A Reflective Terahertz Point Source Meta-Sensor with Asymmetric Meta-Atoms for High-Sensitivity Bio-Sensing
Source: Biosensors (Basel). 2024 Nov 23;14(12):568. doi: 10.3390/bios14120568 (PMC11675057; doi:10.3390/bios14120568)
Supplement: Supplementary file 1 [file biosensors-14-00568-s001.zip › biosensors-3286635-supplementary.pdf]

Supplementary materials for:

A Reflective Terahertz Point Source Meta-sensor with Asymmetric Meta-atoms for High-sensitivity Bio-sensing

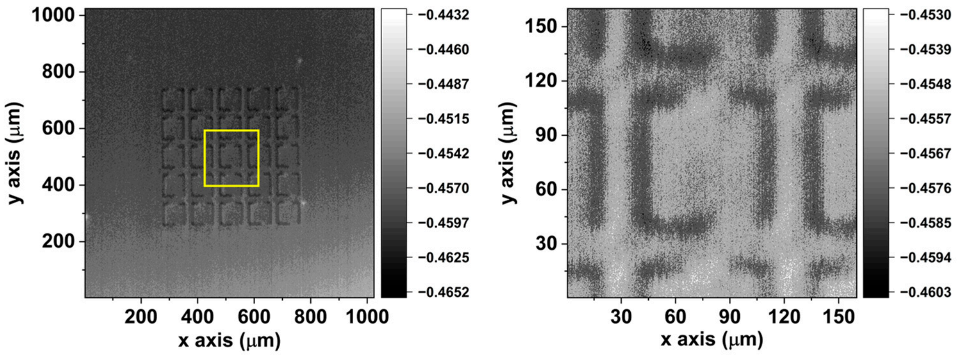

(a) (b)

Figure S1. Laser reflection imaging results of the double-gap asymmetrical meta-structure arranged in a 5 × 5 array with a period of 100 μm. (a) Overall view. (b) Enlarged view of the central meta-atom in the yellow [illegible]

To position the laser irradiation spot, laser reflection imaging was employed. Figure S1 shows the results of the double-gap asymmetrical meta-structure arranged in a 5 × 5 array with a period of 100 μm, including the overall view (Figure. S1(a)) and the enlarged view of the central meta-atom in the yellow box (Figure. S1(b). The laser was focused on the center of the laser reflection image.

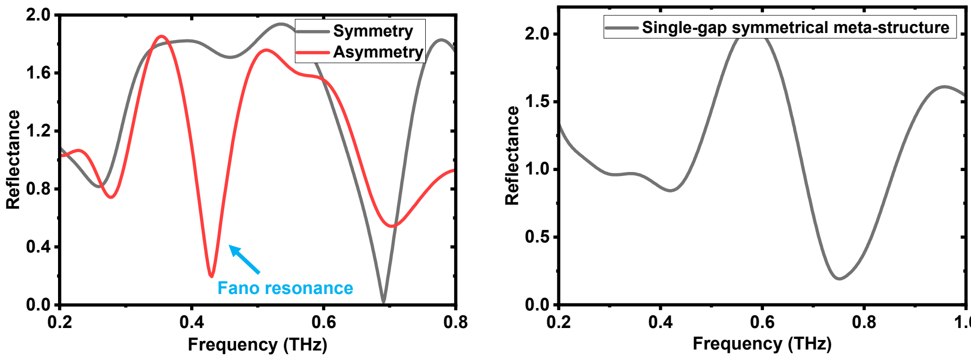

(a) (b)

Figure S2. (a) Reflectance spectra of double-gap symmetrical (D=0) and asymmetrical (D=20 μm) meta-structures. (b) Reflectance spectra of single-gap symmetrical meta-structure.

Figure S2(a) displays the reflectance spectra of double-gap symmetrical (D=0) and asymmetrical (D=20) meta-structures. By breaking the symmetry of the meta-structure [1], a sharp resonance peak appears, which is the Fano resonance, as denoted by the blue arrow. Figure S2(b) shows the reflectance spectra of single-gap symmetrical meta-structure. The array number and period of the three types of meta-structures are set as 5 × 5 and 100 μm, respectively.

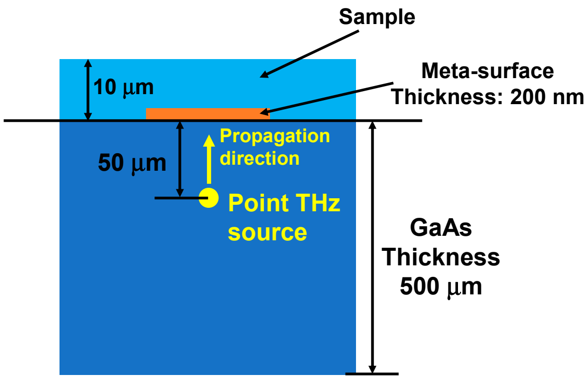

Figure S3. Simulation geometry configuration.

Figure S3 shows the simulation geometry configuration. The thickness of the meta-surface and the GaAs are 200 nm and 500 µm, respectively. The point THz source is placed below the meta-surface with a distance of 50 µm. The thickness of the sample on the meta-surface is 10 µm.

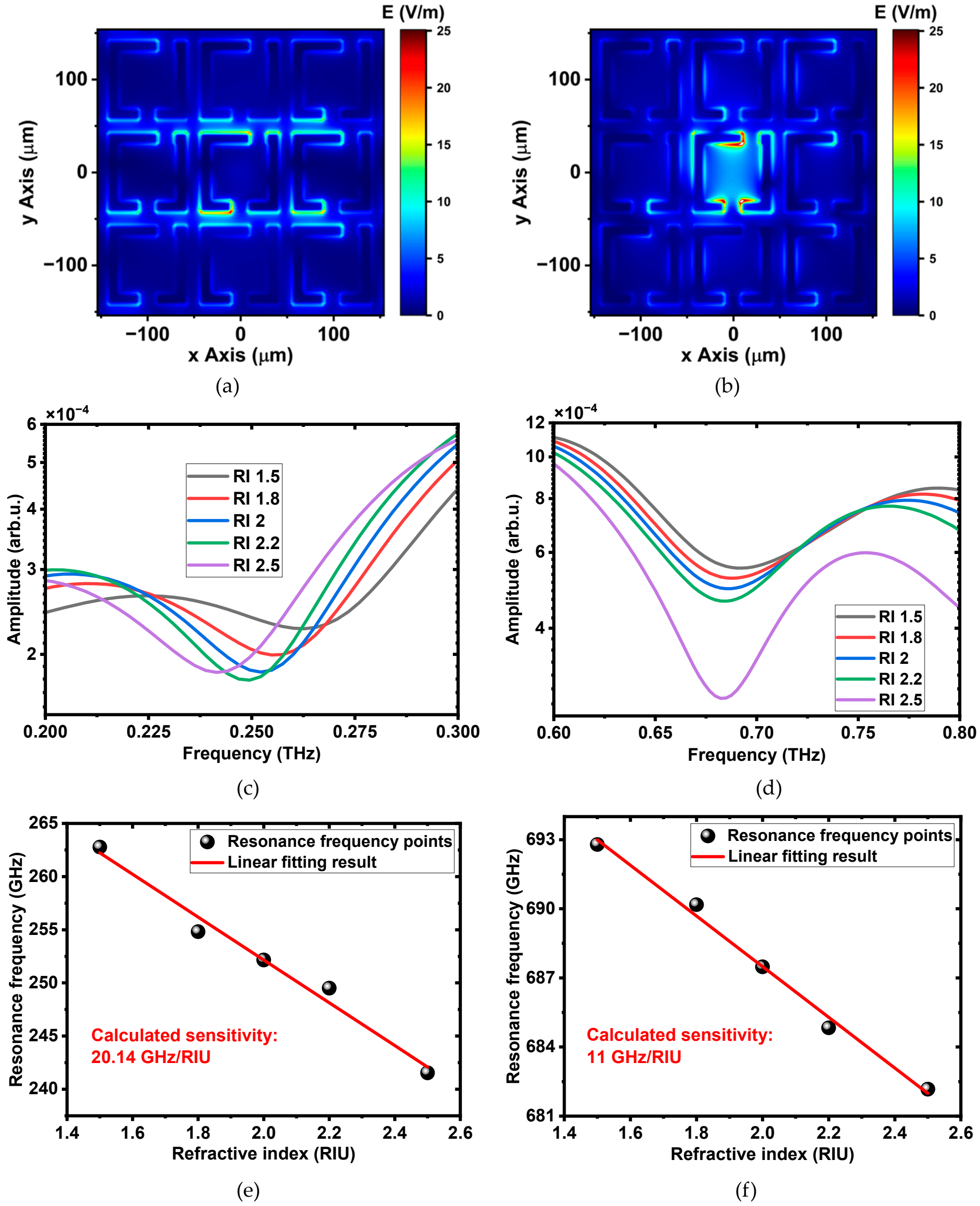

Figure S4. The resonance mode analysis of the 1st and 3rd resonances. The electric field distribution of the 1st and 3rd resonances at 0.275 THz (a) and 0.7 THz (b), respectively. The calculated 1st (c) and 3rd (d) resonance frequency spectra of the meta-structure immersed in materials with different refractive indices. The calculated 1st (e) and 3rd (f) resonance frequency versus refractive index with linear fitting results. The evaluated sensitivity of 1st and 3rd resonances are 20.14 GHz/RIU and 11 GHz/RIU, respectively.

Figure S4 displays the analysis of the 1st and 3rd resonances, including the electric field distribution and the refractive index dependence on the immersed material. Figures S4(a) and S4(b) show the electric field distribution of the 1st and 3rd resonances at 0.275 THz and 0.7 THz, respectively. Stronger electric fields were observed in the coupling region between meta-atoms at 0.275 THz and in the internal region of the single central meta-atom at 0.7 THz. Therefore, the 1st resonance is regarded as a coupling mode, and the 3rd resonance is a resonance mode of the meta-atom itself. To evaluate the sensing performance of these two resonance modes, the refractive index dependence on the immersed material was estimated, as shown in Figures S4(c) and S4(d). As the

refractive index of the surrounding material increases, the resonance peak of both modes gradually shifts to lower values. This is because the material with a larger refractive index increases the floating capacitance of the entire structure, resulting in a decrease in the resonance frequency. Notably, the 1st resonance exhibits a larger frequency shift than that of the 3rd resonance, indicating superior sensitivity. Figures S4(e) and S4(f) show the corresponding sensitivities of the 1st and 3rd resonances, respectively. The black dots are the resonance frequency points extracted from Figures S4(c) and S4(d), and the red lines are the linear fitting results. The calculated sensitivities of the 1st and 3rd resonances are 20.14 GHz/RIU and 11 GHz/RIU, respectively.

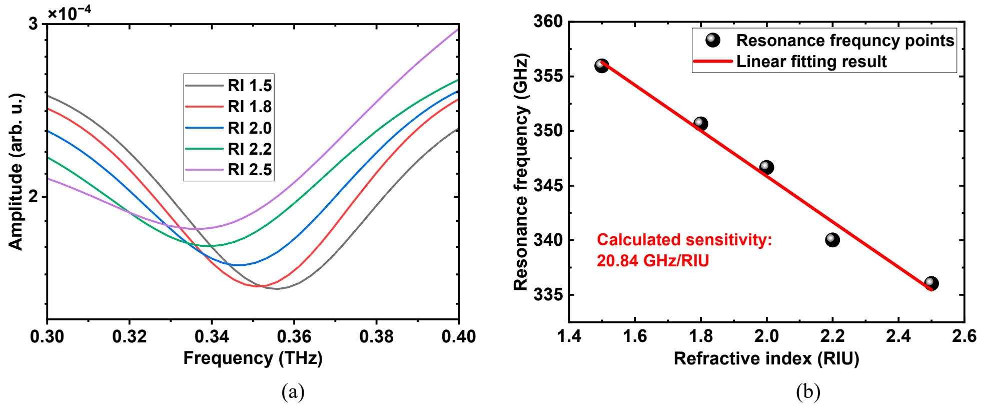

Figure S5. Refractive index dependence of the single-gap symmetrical meta-structure. (a) The calculated resonance frequency spectra of the single-gap symmetrical meta-structure immersed in materials with different refractive indices. The calculated resonance frequency versus refractive index with linear fitting results. The evaluated sensitivity is 20.84 GHz/RIU.

Figure S5 demonstrates the refractive index dependence of the single-gap symmetrical meta structure. In Figure S5(a), the calculated resonance frequency spectra show a gradual shift to lower values as the refractive index increases from 1.5 to 2.5. The resonance frequency points for each refractive index extracted from Figure S5(a) are represented by black dots in Figure S5(b), while the red line indicates the corresponding linear fitting results, with an evaluated sensitivity of 20.84 GHz/RIU.

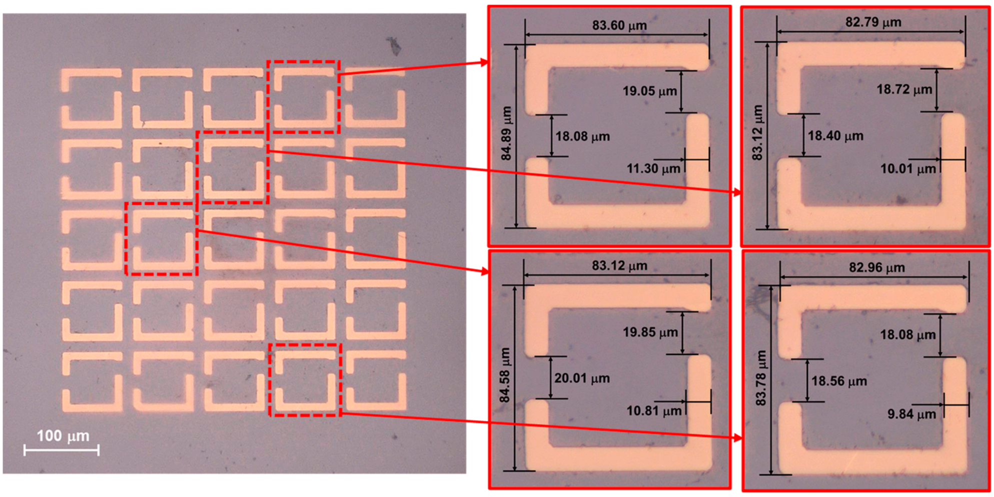

Figure S6. Optical image of the fabricated asymmetrical meta-atoms.

Figure S6 displays the optical image of the fabricated asymmetrical meta-atoms. The left part provides an overall view of the meta-atoms arranged in 5 × 5 arrays with a 100 μm period. The right part demonstrates enlarged views of four individual meta-atom units, along with their respective measured dimensions. Variations exist in the overall length, gap size, and linewidth between the units due to fabrication errors, which ultimately contribute to the discrepancies in the observed resonance frequency response compared to the intended design, as shown in Figure 2(b).

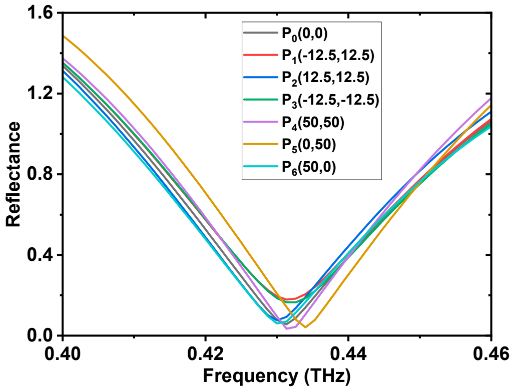

Figure S7. The calculated reflectance spectra at each laser excitation point position.

Figure S7 illustrates the corresponding calculated reflectance spectra at each laser excitation point position. Six points (P_0(0,0), P_1(-12.5,12.5), P_2(12.5,12.5), P_3(-12.5,12.5), P_4(50,50), P_5(0,50), P_6(50,0)) were examined. Slight resonance frequency shift and response amplitude change can be observed. Similar to the experimental results, a larger resonance frequency shift can be obtained when the laser is focused on the coupling area between meta-atoms, compared to the area inside the meta-atom unit.

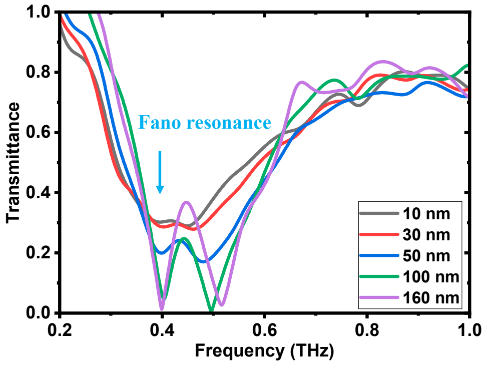

Figure S8. Thickness dependence in the transmission mode. The transmittance spectra of the meta-atom structures with different thicknesses from 10 nm to 160 nm.

Figure S8 shows the thickness dependence measured in transmission mode. The thickness of the structure ranges from 10 nm to 160 nm, which is controlled by the sputtering time. As the thickness decreases, the Fano resonance in the transmission mode [2] becomes gradually weaker, similar to the result in the reflection mode.

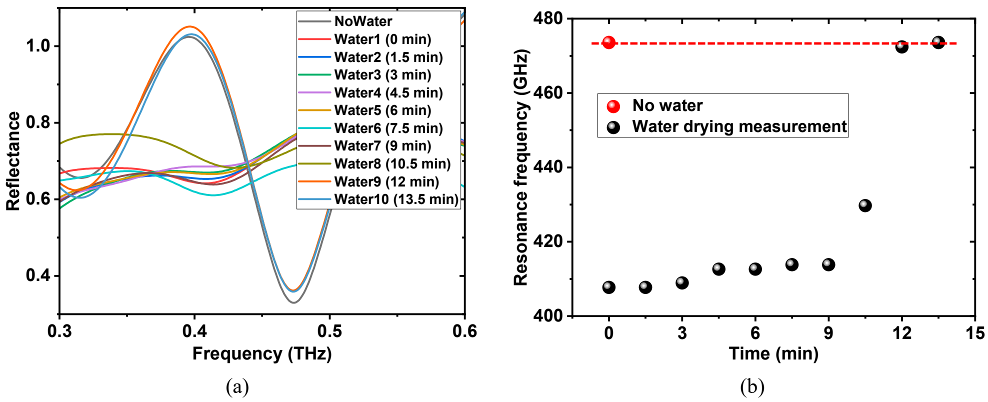

Figure S9. Resonance frequency dependence on ultra-pure water drying process. (a) Reflectance spectra of meta-sensors without and with ultra-pure water in a single measurement. The dropped ultra-pure water was measured 10 times until it completely evaporated. (b) Change in resonance frequency over time (number of measurements) during the ultra-pure water drying process.

Figure S9 illustrates the dependence of the resonance frequency on the water drying processes. In Fig. S9(a), reflectance spectra were obtained from a single measurement to monitor the water drying process. With no dropped ultra-pure water, a resonance peak was observed at approximately 0.47 THz. After dropping ultra-pure water, the resonance frequency was shifted to lower frequencies, and 10 measurements were conducted until the ultra-pure water was completely dry. Each measurement takes 1.5 minutes and it is considered that there is no effect of drying during this time. However, in the DNA measurement performed in Figure 7, the measurement was conducted immediately after dropping the DNA samples. Once the first measurement was finished, the DNA sample was cleaned with ultra-pure water, and it was confirmed that there was no change in the original resonance. This drop-clean process was repeated 10 times, and the averaged reflectance was calculated to enhance the reliability. Fig. S9(b) shows the changes in the resonance frequencies extracted from the spectra in Fig. S9(a). Initially,

the drying process had little effect on the change of the ultra-pure water, which kept the resonance frequency constant. Subsequently, the shape of the ultra-pure water drop changed upon drying, resulting in changes in the resonance frequency. When the ultra-pure water is completely evaporated, the resonance frequency returns to its original state, the same as that before dropping the ultra-pure water.

References

[1] Singh, R.; Al-Naib, I. A.; Koch, M.; Zhang, W. Sharp Fano resonances in THz metamaterials. *Opt. Express* **2011**, 19, 6312-6319.

[2] Serita, K.; Murakami, H.; Kawayama, I.; Tonouchi, M. A terahertz-microfluidic chip with a few arrays of asymmetric meta-atoms for the ultra-trace sensing of solutions. *Photonics* **2019**, 6, 12.
